# Imagination as a fundamental function of the hippocampus

**DOI:** 10.1098/rstb.2021.0336

**Published:** 2022-12-19

**Authors:** Alison E. Comrie, Loren M. Frank, Kenneth Kay

**Affiliations:** ^1^ Neuroscience Graduate Program, University of California San Francisco, 675 Nelson Rising Lane, San Francisco, CA 94158, USA; ^2^ Kavli Institute for Fundamental Neuroscience, University of California San Francisco, 675 Nelson Rising Lane, San Francisco, CA 94158, USA; ^3^ Center for Integrative Neuroscience, University of California San Francisco, 675 Nelson Rising Lane, San Francisco, CA 94158, USA; ^4^ Departments of Physiology and Psychiatry, University of California San Francisco, 675 Nelson Rising Lane, San Francisco, CA 94158, USA; ^5^ Howard Hughes Medical Institute, University of California San Francisco, 675 Nelson Rising Lane, San Francisco, CA 94158, USA; ^6^ Zuckerman Institute, Center for Theoretical Neuroscience, Columbia University, 3227 Broadway, New York, NY 10027, USA

**Keywords:** hippocampus, imagination, place cells, replay, theta, memory

## Abstract

Imagination is a biological function that is vital to human experience and advanced cognition. Despite this importance, it remains unknown how imagination is realized in the brain. Substantial research focusing on the hippocampus, a brain structure traditionally linked to memory, indicates that firing patterns in spatially tuned neurons can represent previous and upcoming paths in space. This work has generally been interpreted under standard views that the hippocampus implements cognitive abilities primarily related to actual experience, whether in the past (e.g. recollection, consolidation), present (e.g. spatial mapping) or future (e.g. planning). However, relatively recent findings in rodents identify robust patterns of hippocampal firing corresponding to a variety of alternatives to actual experience, in many cases without overt reference to the past, present or future. Given these findings, and others on hippocampal contributions to human imagination, we suggest that a fundamental function of the hippocampus is to generate a wealth of hypothetical experiences and thoughts. Under this view, traditional accounts of hippocampal function in episodic memory and spatial navigation can be understood as particular applications of a more general system for imagination. This view also suggests that the hippocampus contributes to a wider range of cognitive abilities than previously thought.

This article is part of the theme issue ‘Thinking about possibilities: mechanisms, ontogeny, functions and phylogeny’.

## 1. Introduction

The ability to imagine is essential to human experience. At a broad level, imagination has a major role in human creativity, agency and everyday thoughts and actions. More specifically, humans have and express many types of imagined experiences. These include recollections, predictions, simulations, counterfactuals, fantasies, suppositions and mind-wandering—and, in pathological cases, hallucinations and confabulations. These wide-ranging forms of imagination are relevant, if not essential, to a similarly wide range of cognitive domains, such as memory, planning, learning and inference. Despite this fundamental importance, our understanding of how imagination is realized as a biological process in the brain remains nascent. Indeed, the sheer diversity of imagined experiences makes it challenging to begin to envision a possible biological approach.

As starting point, we identify a unifying characteristic of imagined experiences: they do not refer to actual present experience, or directly reflect ongoing circumstances in the external world. Rather, imagined experiences refer to non-actualities, and arise from a source internal to the subject. Awake healthy subjects can, in other words, ‘mentally’ self-generate thoughts and experiences and distinguish them from thoughts and experiences driven by ongoing stimuli in the actual present. We refer to this fundamental ability to generate possibilities that do not correspond to the actual present as *generativity*. By this definition, generativity is a basic function that underlies imaginative abilities broadly, regardless of more specific properties, such as references in time (e.g. remembering the past or simulating futures). As further clarification, we also note that our present use of ‘generativity’ differs from its senses in linguistics and in statistical models (notwithstanding potential connections between these uses [1–3]). Defining generativity enables us to focus on a single characteristic ability that may ultimately facilitate our understanding of the diverse types and components of imagination.

Crucially, generativity can be understood at the level of the brain. Mirroring the subject-level ability to distinguish actual from imagined experience [4], specific neural processes in the healthy brain must ‘parse’ internal representations as ongoing experience (actual) versus internally generated alternative experience (imagined). Importantly, this substrate-level generativity does not presuppose features such as mental imagery, mental time travel or conscious awareness. Indeed, defining generativity enables us to refer to the brain's capacity to internally generate experiences that are distinguished from externally driven present experience, without invoking these features that are associated with subjective human imagination. As an example, a soccer player approaching a moving ball can rapidly assess numerous dynamic ongoing events and stimuli, consider multiple possible responses, and decide on a play, all in a split second and without overt awareness of each internally represented possibility. In animals, ethologically relevant scenarios such as predation and escape make similar demands on cognition [5]. Thus, direct investigation of the brain may be essential to understand generativity.

In this review, our overall aim is to describe and advance our understanding of how generativity—an ability underlying imagination—is realized in the brain. Our review is guided by five questions: (i) where generativity might be implemented in the brain, (ii) how generative neural activity can be identified, (iii) what candidate generative neural activity patterns and representational correlates have been previously described and (iv) how the brain can organize actual versus generative activity patterns. This discussion establishes that the hippocampus, a brain structure in the medial temporal lobe, is a candidate biological substrate of generativity, and that patterns of hippocampal neural firing reflect generative processes by representing a diverse range of alternatives to ongoing experience. Finally, we consider (v) what these observations suggest about the biological basis of generativity and its role in cognition. More specifically, in light of recent findings at the level of neuronal firing patterns in rodents, in addition to brain research related to imagination in humans, we suggest that the hippocampus—often understood as a system that characteristically represents actual experience, whether in the past, present or anticipated future—may be better understood as a system that also represents imagined alternatives to actual experience.

## 2. The hippocampus as a locus of generativity in the brain

What structure within the brain might implement generativity? One approach to this question is to determine whether damage to specific parts of the brain causes deficits in imaginative abilities relying on generativity, including recollecting the past, envisioning the future or constructing fictional scenarios. Notably, the earliest case studies linking imagination of the future to specific brain areas are in individuals with previously established deficits in memory of the past [6–10]. In one classic case, patient H.M. suffered severe amnesia after his hippocampus and adjacent medial temporal areas were surgically removed, which established the hippocampus as an important site for memory, particularly episodic memory [11,12]. Notably, while episodic memory impairments are most traditionally reported, H.M. and many other patients with hippocampal damage have since been examined and found to have severe impairments in future-oriented thinking and constructing fictional events more generally [9,13–18]. These findings raise the possibility that recollection of the past, anticipation of the future and imaginative abilities more broadly may share common underlying functions as well as dependence on the hippocampus [17,18].

Complementing lesion studies, functional brain imaging has revealed activation of the hippocampus during a variety of self-reported imagined experiences that overtly differ from subjects' actual circumstances [19–22]. In such studies, subjects are typically asked to imagine experiences that differ from present experience through changes in time, space and/or personal perspective. The hippocampus, in addition to a group of cortical areas known as the default mode network, is consistently activated during, for instance, recalling autobiographical experiences, imagining anticipated future episodes, imagining counterfactuals, mentally simulating common activities (e.g. brushing teeth), constructing fictional scenes, imagining non-actual events and stories, taking on others’ perspectives and unprompted mind-wandering [19,20,23–27]. These results highlight that the hippocampus, along with other brain regions in the default mode network, is important for the capacity to generate mental displacements from actual present circumstances, whether in time, space, personal perspective and possibly other domains [14,17,19,28,29]. Thus, although the cognitive role of the hippocampus is often conceptualized in relation to prior experience (i.e. episodic recollection, recall) or explicitly anticipated experience (i.e. planning, prospection) [30–32], the hippocampus appears to play a more general role in imaginary experience [29].

In efforts to clarify this role, studies have often probed the availability and character of mental imagery. Several further studies help refine the role of the hippocampus beyond the observation mentioned above that hippocampal damage is associated with deficits in vividly visualizing fictional scenes. First, patients with partial hippocampal lesions show activation of residual hippocampal tissue when tasked with imagining complex scenes [33,34]. Second, one patient with longstanding hippocampal damage found it effortful but possible to visualize single imaginary objects and simple scenes, yet could not readily imagine complex scenes in one automatic and coherent picture—instead, he built up the scenes ‘bit by bit’ [33]. Residual hippocampal tissue in this patient was not activated during these tasks as it was in control participants [33]. These findings suggest that the hippocampus is not strictly required for mental imagery, and therefore that the role of the hippocampus in imagination may be only indirectly related to mental imagery. The requirement of the hippocampus for readily constructing complex scenes in particular suggests a different basis or principle by which the hippocampus contributes to imagination [33]; we revisit this issue in the section ‘Generativity as a function of the hippocampus’.

The above lesion and functional imaging work implicates the hippocampus as a candidate substrate for generative thinking, typically by relying on conscious verbal or behavioural reports. This approach is, however, limited in addressing how generative processes are implemented at a neuronal level. For example, the timing of underlying processes relative to eventual behavioural reports remains unclear. Generative processes may also unfold at timescales considerably faster than behaviour, which suggests the need for complementary approaches with finer temporal resolution. Here animal models provide an important advantage by enabling greater access to neural firing. This potential approach in turn raises the question of whether animals also exhibit behaviours indicating generative thought, and if so, whether the hippocampus is also implicated, as in humans.

From work dating at least a century, it is clear that animals behave based on memory of prior experience and conceptual insight rather than solely trial and error, instinct and presently sensed information [35–37]. This implies a corresponding ability to construct and use internal representations and suggests the existence of generative neural processes in animals. In the case of rats, a common model for hippocampal studies, a seminal example of behaviour based on internal representations is spatial navigation. When navigating, rats can take novel paths (for instance, shortcuts to goal locations), implying an internal model enabling the ability to generate such novel courses of action [38,39]. Rat behaviour can also appear deliberative and regretful, suggestive of internally generating representations of possibilities, including counterfactual pasts [40–42]. In service of these and other behaviours, the hippocampus is thought to be essential for using an abstract internal model, or ‘cognitive map’ that relates items, events and features of experience [42–44]. Indeed, hippocampal damage impairs various behaviours thought to rely on abstract internal representations such as rats' abilities to infer relationships between stimuli [45]. Further, hippocampal lesions impair rats’ abilities to make choices dependent on an internal model and predictions or plans made by that model [46]. These findings suggest that the hippocampus is an important locus in the rodent brain for constructing abstract mental models, which in turn could be used to generate representations of prior, new and otherwise not presently experienced possibilities, enabling insightful behaviours.

With the hippocampus as a starting point for investigating generativity in both humans and animals, we now aim to clarify what neural firing patterns have been observed in the hippocampus and what internal representations they suggest. To do so, it is necessary to address our second question: how can generative neural activity patterns be identified?

## 3. Identifying neural firing patterns that are generative

**Figure 1.**
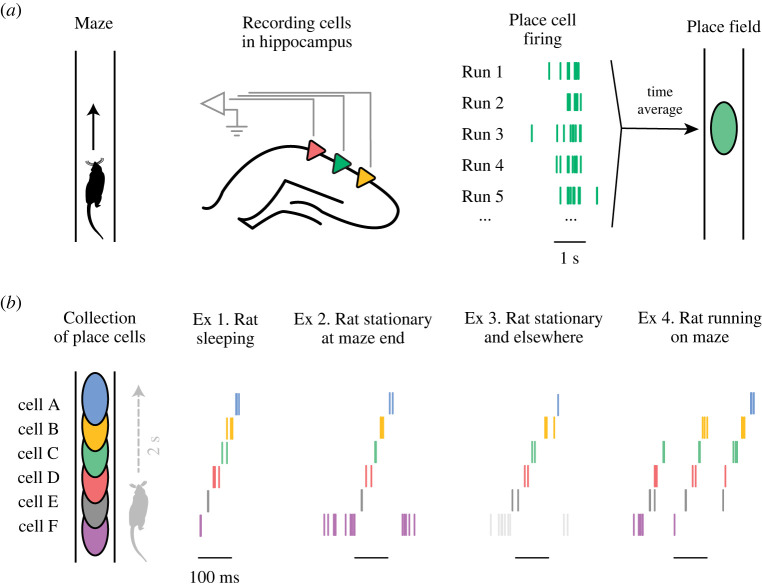
Hippocampal neural firing patterns schematic. (*a*) Place cell firing. Left: a rat runs on a linear maze. Middle: the firing activity of multiple neurons is recorded simultaneously from the hippocampus while the rat runs. Right: one example recorded place cell fires in the same spatial location on the track over many runs, but with notable variability across individual runs (firing denoted by raster lines). The time-averaged firing of the example place cell over many runs forms the cell's place field (oval). (*b*) Generative place cell firing examples. Left: place field locations (ovals) of multiple place cells (A–F) that fire as the rat runs along the linear maze (one run takes approx. 2 s). Right: examples (ex 1–4) of generative firing patterns that occur when the rat is not actually in the cells' place field locations. Place cell firing is denoted by cell-coloured raster lines. Ex 1: replay occurs while sleeping in an environment separate from the maze. Ex 2: replay occurs while rat is stationary at the lower maze end. Ex 3: replay occurs while rat is stationary and in a different maze environment (light grey lines indicate firing of a cell active in the different maze, but not active on the maze shown at Left). Ex 4: occurs while the rat is running on the maze, during the theta rhythm. In ex 1–4, place cell firing corresponds to a series of locations not presently occupied by the rat. (Online version in colour.)

Identifying neural firing patterns that may represent imagined experiences requires us first to identify neural firing that corresponds to actual experience. Here, we focus on studies of neural firing in the rodent hippocampus. To investigate internal representations at the level of neurons, neurobiologists have leveraged the well-established relationship between spatial location and hippocampal firing in freely moving rats [47]. Over 50 years of work have established that principal neurons in the rodent hippocampus exhibit increased firing rates when the animal is in distinct physical locations (figure 1*a*) [47,48]. As the rat moves through an environment, each of these ‘place cells’ consistently increases its firing rate when the animal is in the neuron's ‘place field’ location(s) [47,48]. Importantly, place cell firing also varies based on numerous factors besides location [49]; for example, in linear environments, a large proportion of place cells fire more when the animal is travelling in a particular direction [50]. Therefore, at a broader level, it is important to note that a place field describes average firing over many individual runs through a location, even though there is often substantial variability in a place cell's firing across individual runs through the same place (figure 1*a*).

The basic notion of a place field, along with the ubiquity of place cells in the rat hippocampus, provides a possible approach to identifying actual and generative activity at a neural level. If we take a place cell's activity to represent its place field location, then each instance of firing by that neuron can be provisionally understood as representing that location. By this interpretation, a place cell will reliably fire when the animal is in the cell's place field, thereby representing the animal's actual present location.

Importantly, in certain moments, a place cell can also fire when the animal is not actually in the cell's time-averaged place field location (figure 1*a*,*b*) [51–53]. Accordingly, these moments can be provisionally understood as times in which a representation of the place field location is internally generated, even though the animal actually occupies a different location at that moment.

Strikingly, place cells have been found to fire outside of their place fields in coordination with each other (figure 1*b*) [54,55]. During these events, the collective activity of place cells can be understood to express a representation that corresponds to locations different from the animal's current location [52,53]. In other words, this neural firing is consistent with a generative representation; while it appears displaced from the animal's actual state and present stimuli, it is internally coordinated across cells (figure 1*b*).

A variety of analysis methods have been used to investigate these generative firing events and internal spatial representations in the hippocampus [56–58]. Briefly, one approach is to model the firing of many individual place cells as their time-averaged place field locations, and then invert that model to produce an estimate of the neurally represented location at each moment in time [59–61]. Doing so enables us to infer, or decode, the animal's moment-to-moment ‘mental location’ based on hippocampal firing patterns. Thus, by identifying periods when the decoded representation of location (or direction) differs from the animal's actual state, we can examine periods when hippocampal activity is collectively inconsistent with a representation of actual experience and may instead be generative. This enables us to address our third question: what kinds of generative representations have been observed in the hippocampus?

## 4. Generative representations in hippocampal neural firing

Single-cell and population decoding approaches have revealed a striking variety of putative generative representations in the rat hippocampus over the past several decades [62–65]. Traditionally, these representations have been accounted for as specific episodes and abstracted experiences that are based on the past, or that anticipate experiences in the future [66,67]. Recent results, however, imply that the hippocampus also regularly represents alternatives to actual experience, whether in the past, present or anticipated future [68–70]. Together, these findings suggest that the hippocampus may generate a substantially wider range of internally constructed alternatives to the animal's actual experience than traditionally understood.

### (a) Representations consistent with past experiences

The first reports of hippocampal activity patterns related to past experiences focused on sleep [51,54]. Firing sequences of place cells that were active during running on a maze were found to reactivate in similar sequential order during subsequent sleep, as if briefly ‘replaying’ past spatial experience [71–73]. These replays occur on the order of tens to hundreds of milliseconds, far faster than the seconds-long timescale over which the actual behavioural traversal of those locations unfolds (figure 1*b*) [71]. Importantly, replay events were subsequently found also to occur during waking periods in which rats are behaviourally immobile, such as sitting still or eating (figure 1*b*) [74,75]. During wake and sleep, replay typically occurs during a burst-like hippocampal network-level activity pattern, the sharp wave-ripple (SWR), that is itself internally generated (rather than externally driven), consistent with the notion of generativity [76].

As suggested by its name, replay has been interpreted as recapitulating specific episodes of prior experience. An early observation was that after an animal ran towards and then came to rest at a reward location, a path was replayed starting at the animal location and proceeding in reverse, as if retracing the path that led to the reward [75,77,78]. Replay representations not only initiate at a stationary animal's location [74], but can also correspond to paths that start farther away from the animal within the current maze, as well as on a different maze experienced beforehand (figure 1*b*) [79,80]. These examples are evocative of the hippocampus' long hypothesized role in cognitive functions that rely on experiences from the past, such as memory consolidation and episodic recall [65,81].

Additional findings on replay suggest a more complex picture. Unlike a rigidly recapitulative process that uniformly represents recent experiences, replay can be enriched for previously taken paths associated with reward, paths associated with aversive outcomes, nearby locations and paths that have not recently been taken [61,82–84]. Further, these and several additional findings [82,84–88] suggest that replay events are collectively well described as reflecting an abstract internal spatial model of the encountered environment, or a spatial ‘cognitive map’ [43,52,62]. For instance, replays can be biased toward paths that are less behaviourally traversed, and replays can be consistent with random trajectories through a familiar space [87,88]; replays like these may sample locations that are not the most behaviourally salient or the most physically occupied to support the maintenance of a flexible model of the environment, and this function could help explain why replays are inconsistent with a rigid recapitulation that passively records recent experience [84,87,88]. These reports suggest that replay, instead of directly reinstating specific episodes, may abstractly reflect past experience via an internal spatial map.

While there is little doubt that replays can be derived from prior experience, both in the case of a rigid recapitulation or abstract model based on the past, what remains unclear is whether neural processes within or beyond the hippocampus interpret replay events as temporally situated in the past. For example, a replay of recently traversed locations behind the animal, that are not subsequently traversed, is better correlated with past than future behaviour, but this does not rule out the possibility that this replay represented a potential future traversal of those locations, or a spatial sequence without a projection in time. Despite this ambiguity, replay can indeed be related to prior behavioural experiences. And, moreover, these findings on replay exemplify how generative activity in the hippocampus can represent various possibilities that differ from the actual present—here, in the form of spatial paths in known environments.

In parallel to replay during rest, neural firing in the hippocampus during movement has also been suggested to be recapitulative. During movement, an internally generated network-level activity pattern, the 8 Hz theta rhythm, is observed throughout the rodent hippocampus [89–92]. Place cells are known to fire systematically in relation to the theta rhythm, such that neurons with place fields behind, at and ahead of the animal fire at early, intermediate and later phases of theta cycles, respectively [55,93,94]. Accordingly, collective place cell firing during a single cycle can represent a series of locations consistent with sweeping from the immediate past and present ahead to anticipated future locations (rightmost example in figure 1*b*) [63]. Although firing in early phases of the theta rhythm can recapitulate locations just traversed by the animal, this firing appears to be consistent with the immediate actual past (for instance, as opposed to alternative past (counterfactual) locations) [63,95]. This suggests that early theta phase representations may also be best understood as reflecting actual experience, and not possible experience. That said, hippocampal firing during movement can correspond to locations behind the animal and is often thought to reflect the recent past [53,96,97].

### (b) Representations consistent with anticipated futures

**Figure 2.**
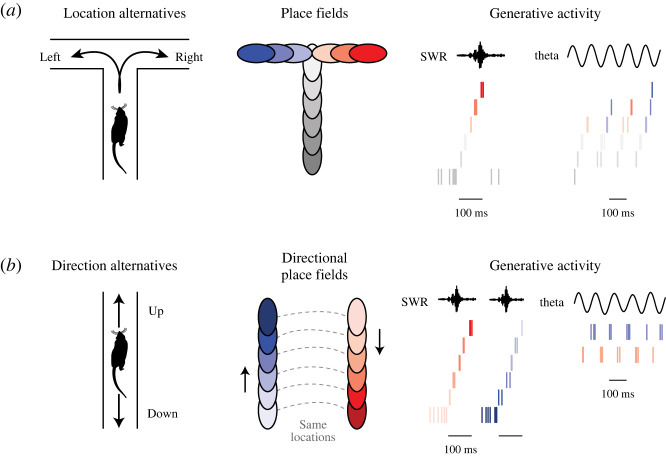
Generative activity corresponding to alternative possibilities. (*a*) Example of neural firing corresponding to alternative locations. Left: a rat running on the central arm of a bifurcating maze can either turn left or right. Middle: individual place cells fire when the animal is in particular place field locations on the maze (ovals coloured by cell). Right: generative activity. While the rat is resting at the end of the central maze arm, generative neural firing during a sharp wave-ripple (SWR) can replay other locations (raster lines coloured by cell). At other times while the rat is running up the central maze arm towards the bifurcation, neural firing during the theta rhythm alternates between current and upcoming locations within each cycle, and between the left and right trajectories ahead across the second halves of theta cycles. (*b*) Example of neural firing corresponding to alternative directions. Left: a rat on a linear maze can run in up or down directions. Middle: different sets of direction-selective place cells fire in their place field locations (ovals) when the rat runs up or down (ovals coloured by cell). Right: generative activity. Neural firing during SWRs replays locations that do not correspond to the rat's actual location at the end of the maze. Additionally, while the rat is running in one direction, cells corresponding to the rat's actual (red) and alternative (blue) directions fire in alternation along the theta rhythm. (Online version in colour.)

Place cell firing can also correspond to upcoming spatial paths, suggesting that generative representations may anticipate future experience. As introduced above, place cells firing in late phases of theta cycles tend to have place fields in locations ahead of the animal [53,55]. The extent to which this activity projects ahead of the animal can correlate with the distance the animal subsequently traverses, consistent with the possibility of future anticipation or prediction [98]. When multiple paths are available (such as a path bifurcating), hippocampal firing has been found to proceed ahead along only one path at a time [68,99]. Furthermore, place cell firing corresponding to the left or right path ahead can occur on interleaved theta cycles, consistent with serially representing alternatives (figure 2*a*) [68]. These internally generated representations are consistent with generatively representing anticipated possibilities, and are reminiscent of deliberation [99]. However, while in some cases theta-associated neural firing can predict the animal's subsequently taken path [99–101], firing patterns associated with alternation between paths fail to reliably predict the animal's subsequent choice [68,99].

Apart from generative activity associated with theta, replays suggestive of anticipated future experience have also been reported. In early work, replay was found to correspond to sequences of locations starting near and projecting ahead of the animal, just prior to running along that same path in the linear maze, consistent with anticipation of upcoming experience [74,79,80]. Since then, several studies have reported that replay in environments with more options (an open arena or multi-arm maze) is biased toward goal locations that the animal subsequently visits [102,103]. While replay can indeed correspond to subsequently taken paths, recent work from our group shows that replay fails to predict upcoming choices [82].

Seeking to relate generative firing to behavioural episodes in subjects' past or future (e.g. the choice of maze arm in the previous or next trial) has been a common approach in investigating the contributions of hippocampal activity to cognitive functions, especially past-oriented functions such as episodic recall and future-oriented functions such as planning. Task paradigms that disambiguate prior from upcoming experience are well suited for this approach [82]. However, relating generative neural activity to particular locations behaviourally occupied in the past and future does not necessarily indicate that such activity is an internal representation that refers temporally to the past or future. For example, neural firing corresponding to one of two paths ahead of the subject is consistent with a possible future, yet may also reflect recall of a prior traversal of that location, or simply not have any reference in time. In this sense, it remains an open question whether generative firing patterns observed in the hippocampus can refer to experiences projected into the future. Apart from this, it remains the case that some instances of generative firing during theta and replay can correspond to potential future locations, and may thereby contribute to explicitly anticipatory functions such as planning.

### (c) Representations consistent with alternative possibilities

Firing patterns corresponding to locations different from a subject's actual location indicate that the hippocampus can generate representations of alternatives to actual ongoing experience. As discussed above, it has been hypothesized that these firing patterns reflect internal representations referring to episodes of experience in the past or anticipated future. Critically, recent findings indicate that generative firing patterns exhibit properties that may be more consistent with an underlying process that generates representations of non-actual hypotheticals and possibilities more broadly, rather than a process characterized primarily by projection of actual experience in time [68–70].

In recent work focusing on periods of movement, we found that neural firing in the rat hippocampus can regularly represent various alternatives with striking speed and regularity (figure 2) [68]. In initial observations, we found that alternative locations ahead of moving animals could be represented not only as quickly as the frequency of the theta rhythm (approx. 125 ms cycles), but also sustained across many consecutive theta cycles (figure 2*a*) [68]. As in previous work showing that place cell activity can serially alternate between upcoming paths [99], or correspond to paths subsequently taken [98,102], one possibility is to interpret this pattern of neural firing as reflecting an essentially anticipatory function, such as planning or deliberating over future behaviour.

However, we also found that place cell firing corresponding to opposite directions of travel exhibited the same pattern of serial alternation: sustained 16 Hz cycling between the animal's actual direction and an alternative, or non-actual, direction (figure 2*b*) [68]. Toward clarifying what this pattern of alternating activity might reflect about the underlying process in the hippocampus, we highlight three points of consideration.

First, this generative firing pattern has no overt or intuitive temporal reference. Unlike the case of alternative locations ahead of the animal, alternative direction is neither more consistent with upcoming experience, nor more consistent with previous experience. This was especially the case given the experimental setting, in which rats routinely travelled in either direction through a maze as part of navigating in an alternation task [68] (similar to figure 2). Thus, neural firing signalling the non-actual direction was just as plausibly a recollected past as an anticipated future. Importantly, this ambiguity regarding time extends further: it is also just as plausible that the firing pattern reflected a representation of a counterfactual past, an alternative present or an experience with no specific reference in time. This last possibility is reminiscent of imaginative thoughts in humans which do not explicitly project experience into the past or future, but nonetheless differ from a subject's present circumstances. Without further knowledge, it may be relatively parsimonious not to attribute temporal reference to the observed hippocampal firing pattern—rather, a simpler interpretation is that this neural activity corresponded to non-actual experience.

Second, the speed of alternations between actuality and location or direction may be at odds with conscious human thought processes that are, at least subjectively, slower than approximately 125 ms theta cycles. For this reason, we speculate that a neural process at this speed is unlikely to be directly coupled to conscious awareness, such as during a human subject's internal deliberation over two choices, or mental imagery of a remembered episode of navigating a path. Rather, these generative neural firing patterns suggest a function that, like generativity, is marked by moment-to-moment variability and productivity.

The third point is that this generative hippocampal activity, which alternated between possibilities not actually being presently experienced, was largely independent from behaviour [68]. This was the case both for cycling of non-actual locations and direction. Specifically, generative alternating firing patterns occurred commonly across classes of locomotor behaviours (e.g. running, crawling, turning, head scanning, brief pauses in running). Additionally, the number of theta cycles corresponding to alternatives varied widely between instances of otherwise similar trajectories through the maze. Further, activity that cycled between to two paths ahead at a bifurcation did not reliably predict animals' upcoming turn behaviour on individual run trajectories [68]. These observations suggest an underlying process that can be uncoupled from behaviour at three levels: classes of behavioural state, behavioural trajectories and upcoming behavioural choices.

**Figure 3.**
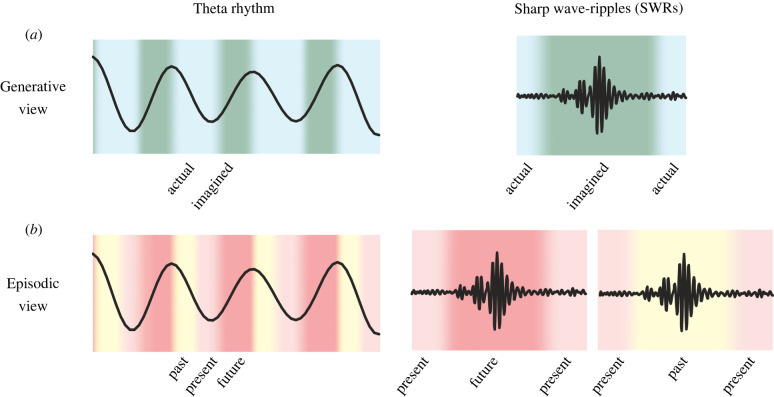
Two views of hippocampal firing during network-level activity patterns. (*a*) Generative view (proposed). Neural firing during early theta phases represents actual circumstances, while representations during late theta phases and sharp wave-ripple (SWR) replays are imagined (generative). (*b*) Episodic view (traditional). Neural firing during each theta cycle corresponds to sequential locations in the past, present and future, and neural firing during SWR replays correspond to past or future locations. Note that the episodic view can be understood as a particular application of the proposed generative view. (Online version in colour.)

These three points are complemented by an additional line of investigation. Examination of the timing of neural firing within theta cycles revealed a surprising commonality between firing corresponding to alternative locations and direction: for either representational correlate, firing corresponding to the non-actual circumstance occurred specifically in late phases of theta cycles (figure 2*a*,*b*) [68]. This observation indicates that late theta phases, previously understood to contain firing related to upcoming paths [53,67], are not exclusive to locations ahead of the animal (figure 3). Rather, late phases can also contain firing related to alternative direction, which raises the possibility that firing corresponding to alternative possibilities in any other domain encoded by the hippocampus may also be generated in late theta phases. Toward this, further observations from our group suggest two additional examples of generative firing during late phases of theta; firing may correspond to locations behind the animal on a path that was not just taken, suggesting an alternative past representation [68], and may also correspond to locations relatively far from the animal, not only locations immediately ahead [70]. Together, these findings illustrate that late-phase firing can correspond to multiple kinds of alternatives to actual ongoing experience (direction and various locations). This is surprising because it is not consistent with the canonical understanding of theta cycles as organizing a sequential representation of locations along a single path, sweeping from past to future locations, or behind to ahead of the animal in space and time (figure 3*b*) [53,55,67,94]. Rather, these findings suggest revising the established view that hippocampal firing during late theta phases corresponds to locations immediately ahead of the animal. A more inclusive view is that late theta phases may be enriched for firing related to a diversity of alternative possibilities and hypotheticals, including, but not limited to, anticipated experiences (figure 3*a*).

**Figure 4.**
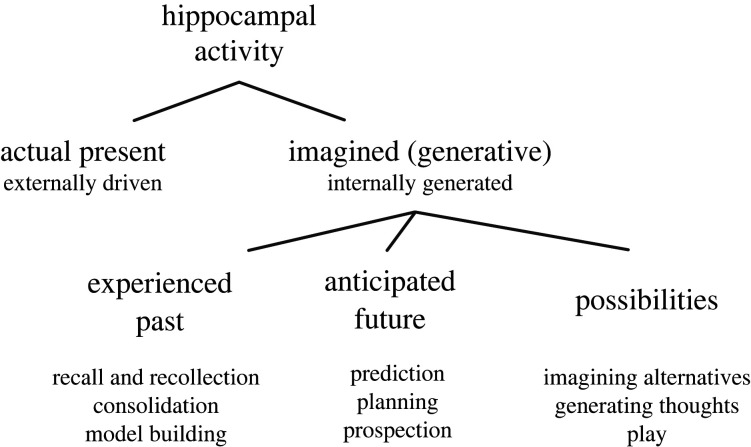
Schema for interpreting hippocampal activity. We suggest that generative activity arising from the hippocampus may not only correspond to the experienced past or anticipated future, but also to a wide range of possibilities. This view may also organize or suggest various cognitive functions.

In the preceding discussion, we largely focused on recent findings regarding hippocampal neural activity associated with movement. Several parallel results indicate that replay events, occurring during periods of immobility, can also represent possible or hypothetical experiences that are not clearly recapitulative nor anticipatory. Replays can represent trajectories that link physically connected spatial paths that the animal has not traversed behaviourally, as if simulating short-cut paths [84,104]. Such synthesized trajectories were not directly experienced by the animal, and therefore are inconsistent with strict recapitulation of the past. Furthermore, in some cases subjects never took the shortcut paths, suggesting that these replays may not have been anticipatory. Recently, another study found that replay is biased to an unchosen path even when that path would not fulfil the animal's motivational state (i.e. biased to water when hungry, and food when thirsty) [69]. This finding is inconsistent with both replay of the recent past and of the immediate future. In sum, generative neural firing in the hippocampus during both movement (theta) and rest (replay) may reflect a process that represents a diversity of possibilities that are alternatives to actual present experience (figures 3 and 4).

## 5. Organization and origin of generative activity in the brain

Having reviewed multiple types of generative neural activity in the hippocampus, we turn to our next question of how generative representations may be organized and ‘parsed’ from representations of actual, ongoing experience. One would expect that neural processes are in place to separate actual and generative activity to avoid their confusion, reminiscent of the subject-level ability to internally distinguish actual from imagined experience [4]. Multiple organizational schemes are possible; different sets of neurons could participate in actual versus generative representations, these representations could occur at different relative times, or some combination of these schemes could take place.

Findings in the rodent hippocampus indicate that neural firing corresponding to actual and generative representations occur at different relative times that are internally determined [105]. Generative representations tend to occur not only with temporal separation from representations of actuality, but also in alignment with underlying network-level activity patterns in the hippocampus that are internally generated: SWRs and the theta rhythm (figure 3*a*) [68,106]. This results in a serial alternation of neural firing corresponding to actuality and generativity, or a temporal ‘multiplexing’ of actual and generative representations in the brain.

This serial alternation is present across behavioural states. During immobility, neural firing corresponding to the animal's actual present location is maintained for prolonged periods, transiently suppressed during SWR events that typically contain generative replays (tens to hundreds of milliseconds), and then subsequently restored (figure 3) [106,107].

Similarly, during movement and exploratory behaviours, neural firing corresponding to actual present and non-actual alternative experience, or actual and generative representations, occurs serially and in alignment with characteristic phases of the theta rhythm [3,68]. More specifically, early phases characteristically contain representations of the animal's actual past and present experience, while late phases may contain firing corresponding to a variety of hypothetical experiences, resulting in alternating actual and generative representations (examples in figure 2, schematic in figure 3) [68]. Furthermore, there are multiple levels of alternation between actual and generative activity during movement—representations not only alternate within approximately 125 ms theta cycles (e.g. actual and upcoming position), but also across consecutive theta cycles (e.g. alternation of two possible paths ahead; figure 2) [68]. Additional findings are also consistent with the idea that multiple representations can be accommodated in the hippocampus via serial alternation at a sub-second timescale. For instance, studies in the rat hippocampus have reported theta-modulated ‘flickering’ between representations of two environmental contexts, as well as dynamic switching between two spatial reference frames, and separate reverse and forward-ordered location sequences within theta cycles [108–110].

The organization of actual and generative neural firing in the hippocampus also extends to other brain areas, consistent with the engagement of a distributed network in these representations [20,111,112]. Network-level neural activity patterns underlying generative representations can be coherent across the hippocampus and prefrontal cortex during replays and along the theta rhythm, with some reports of concurrent expression of actual versus alternative location representations across both regions [107,113–117]. Additionally, some generative firing events in the hippocampus are not only coordinated with but also predicted by the activity of cells in the medial prefrontal cortex [70]. Numerous other cortical and subcortical areas also share coordinated firing patterns with the hippocampus, during both replay events and the theta rhythm [67,118–125]. Recruitment of a large network of brain areas during activity related to actual and generative experience appears to reflect brain-wide organization, and the question of how firing patterns in other regions across the brain specifically contribute and respond to generative representations in the hippocampus remains an active area of research [113,122].

How might organized generative neural firing patterns in the hippocampus come about through hippocampal and extrahippocampal processes? This remains largely unknown, but some initial points can be made. First, one would expect generative firing patterns, which do not correspond to immediately ongoing circumstances, to arise primarily from internally driven activity patterns, as opposed neural activity driven directly by external stimuli. Consistent with this, generative events are observed during SWRs and in association with the theta rhythm—and both of these activity patterns are generated internally in the brain (spontaneously) rather than elicited by external stimuli [76,126]. More specifically, SWRs spontaneously occur during sleep in the absence of dynamic sensory stimuli and can be intrinsically generated in isolated hippocampal slices *in vitro* [76]. Hippocampal theta oscillations arise *in vivo* in coordination with a rhythm generator region, the medial septum, and can also be generated in isolated rodent hippocampus *in vitro* [127,128]. Furthermore, late phases of theta, during which generative representations tend to occur, are associated with increased recurrent network activity from within the hippocampus, and relatively weaker influence from cortical areas that are thought to provide multimodal information to the hippocampus [63,67,129,130].

While SWR and theta oscillations are understood to be internally generated and are associated with the occurrence of generative neural firing patterns in the hippocampus, the question of how specific groups of neurons (such as place cells with overlapping place fields) are recruited during generative events remains open [131]. In addition to mechanisms that support SWR and theta generation, it is likely the case that input from brain regions beyond the hippocampus have a role in this process [67]. One possibility is that the activation of particular sets of spatially tuned neurons during generative events is guided by extrahippocampal areas, such as the prefrontal cortex, that are also implicated in the default mode network [20]. This possibility is consistent with evidence that cortical activity can predict generative spiking during theta oscillations several cycles in advance, as well as SWR activity during sleep, and would argue against the idea that hippocampal ensembles are activated by exclusively unstructured input [70,125]. Studies focusing on the internal correlates of generative activity within the brain, over external behavioural correlates, may be especially important to understand what determines the generative neural firing patterns observed in the hippocampus.

The segregation of generative and actual representations in the hippocampus also raises the question of whether the hippocampus further differentiates subtypes of generative representations. For example, are events that reflect veridical experience from the past somehow distinguished from those that reflect constructed alternatives, or those that are predictive of future choices? At the level of neural firing, it remains unclear whether or how the hippocampus might separate these possible representations. However, two points of reference in the human literature offer clues that the relevant neural substrates may be outside the hippocampus. First, patients with hippocampal amnesia can entertain thoughts that distinguish the past or the future, despite impairments in episodic memory [132,133]. Additionally, hippocampal activation during mental simulations without temporal placement versus those specifically set in the future result in similar activation levels in the medial temporal lobe and default mode network [132,134]. These results are consistent with the idea that temporally differentiating representations related to the past or the future may not be hippocampally dependent. Second, healthy human subjects can subjectively discriminate internally and externally derived information, an ability known as reality monitoring [135]. Based on functional imaging studies in both healthy subjects and patients with schizophrenia who experience hallucinations, reality monitoring is thought to rely primarily on prefrontal cortical networks [112]. By contrast, another study reports that hippocampal activation was similar across cases of true and false recognition memory [136], further suggesting that this ability does not strictly rely on the hippocampus. Although probing reality monitoring in rodents is not straightforward, it would be notable if, for example, frontal cortical firing patterns systematically differed based on the representation of possibilities in the hippocampus that reflected veridical experience versus constructed alternatives. Such a result would be consistent with the idea that the hippocampus alone may not distinguish subcategories of generative events, but that the brain may do so via the engagement of prefrontal circuits.

Looking beyond rodents, it remains an open question as to which patterns of generative activity in the hippocampus are shared across species [137]. On the one hand, SWRs have been observed in a range of vertebrates, as have neural reactivation patterns suggestive of replay [138–144]. In humans, replay and replay-like patterns have also been reported, including activity patterns consistent with reactivating prior experience, as well as inferred sequential activity that is not simply recapitulative [145–149]. By contrast to the ubiquity of SWRs across vertebrates, the theta rhythm appears to be more prominent and continuous in the rodent hippocampus than in various other species [137]. A notable example is the bat hippocampus, which shows network-level activity fluctuations that are not generally rhythmic yet still organize place cell firing according to phase [140,150–153]. This may suggest that actual and generative representations can be organized via temporal multiplexing even in the absence of strong rhythmicity. In nonhuman primates and humans, the hippocampal theta rhythm appears to occur in intermittent bouts and at a lower frequency [140,150–153]. Recently, theta phase coding has also been shown in single cells in human subjects [154,155]. In all, these results indicate some conservation across species of the organization of neural firing with respect to network-level hippocampal activity. More generally, they leave open the possibility that the brains of many species temporally multiplex actual versus generative internal representations.

## 6. Generativity as a function of the hippocampus

Recent findings described above suggest that the hippocampus regularly generates a wider range of representations than previously thought. What functional implications does this suggest?

The cognitive roles commonly ascribed to the hippocampus offer a starting point. Existing theories of hippocampal function are often based on the established role of the hippocampus in human episodic memory [30–32]. Under an essentially episodic view, hippocampal activity necessarily represents or refers to both to time and space, in accordance with the definition of an episodic experience (figure 3*b*) [156]. Along these lines, hypotheses based on this view propose that hippocampal neural firing corresponds to cognitive processes such as past-oriented memory retrieval and consolidation, or future-oriented planning and prospection [3,65]. The view that hippocampal neural firing can support memory of past episodes has been suggested by findings that causally link SWR events (which generally co-occur with replay) to performance on tasks requiring memory of a choice made on a previous trial [157,158], although the diversity of generative neural firing patterns reviewed above during replays suggests a more complex picture [52,62,65]. Both replay as well as theta-associated generative representations have also been posited as anticipatory processes, such as planning, in support of decision-making [159]. These ideas have been reinforced by observations reviewed above that replay and theta-associated activity can relate not only to past but also potentially upcoming behaviour. To account for findings that neither of these firing patterns appears to encode animals' upcoming choice with high reliability [52,68,82,99] (but see [98,100,101]), another version of the anticipatory planning hypothesis posits that the hippocampus generates a ‘menu’ of relevant options evaluated by other brain areas prior to the decision [41,99].

Each of these functional interpretations is plausible and conceptually important, yet only consistent with a subset of the instances and properties of generative neural firing patterns across studies reviewed above. Specifically, it is unclear how retrieval of past experience and planning for the future account for the prevalence and variety of generative representations observed, particularly those that are ambiguously related or unrelated to behaviour in the past or future, and those that run counter to immediate experiences and choices [68,69,84].

As an alternative view, generative firing patterns may be understood as characteristically expressing alternatives to actual circumstances, irrespective of whether those circumstances are in the past, present or future. Indeed, we suggest that temporal referencing to the past, present or future for a given firing pattern in the hippocampus may not be intrinsic or essential (figure 3*a*), a view which has also been posited to account for recent findings in human subjects [132].

By this interpretation, past- and future-oriented cognitive functions that require or involve the hippocampus would be particular applications of a broader and more essential underlying role in generativity, or representing non-actual experiences including possibilities and hypotheticals. This view of the hippocampus' role at the level of cognition is closer to imagination (figure 4). A neural system implementing generativity may often construct a variety of potentially useful representations that do not necessarily relate to known circumstances, or predict immediate behaviour, yet remain relevant for behaviour in an indefinite horizon of time. This advantage is akin to that of insightful thoughts generated in the course of seemingly undirected mental activity, such as free reflection or mind-wandering.

If generative neural activity in the hippocampus does not intrinsically refer to actual experiences, what type of relevance might it have to experience, thought and behaviour? One unifying idea is that the hippocampus is a system for ‘relational’ memory: a system for inferring abstract relationships between observable events (such as sensory stimuli, actions and internal states). Integrating information in relational memory is beneficial in that it enables inference and generalization to novel circumstances, such as those where elements of previous experience are reconfigured [42,160,161]. This can be advantageous regardless of whether those novel circumstances can be anticipated at the time of generating the relational information, consistent with the idea that generative activity can but does not always relate to immediate behaviour.

Generativity could support relational thinking by bringing together elements from experience that are not actually experienced together. Specifically, the role of generative activity could be to combine otherwise separate elements of experience so that their relationship can be inferred. To illustrate, one classic example is inference of a transitive relationship; if a subject learns that A should be chosen over B and B over C from real-world experiences, then the subject can infer that A should be chosen over C, despite never having experienced A and C together. This sort of inferential ability is dependent on the hippocampus in rodents and is furthermore associated with hippocampal activation in humans [45,162–164]. A second example is inferring spatial relationships, which often relies on the ability to link physically discontiguous prior experiences. Rodent behaviour has long indicated the ability to infer novel routes, including more efficient shortcuts, through a spatial environment [38,39]. At the level of neural firing, replay events can stitch together into one coherent representation two track segments that the rat has never traversed in a single run, and can represent novel paths to goals that have not been taken before [84,104]. Further, the mouse hippocampus has been shown to coactivate neurons related to representation of distinct events during SWRs in an inferential reasoning task [164]. Generative representations such as these appear to combine information across separate prior episodes into internally constructed possibilities that may have the potential, but are not required, to inform behaviour. Consistent with these findings, not only is human hippocampal activation associated with correct inferential choices [164], human subjects also exhibit internally generated sequences of hippocampal activity that reorder elements of experience into novel, inferred sequences that do not simply recapitulate previously experienced sequences [145]. Taken together, these results suggest that generative hippocampal activity may be well suited to contribute to relational thinking, and ultimately the internal generation of new knowledge that goes beyond actual experience.

Importantly, inferences in such a relational memory system can operate not only across various modalities, such as sensory, motor and internal states, but also generate all kinds of relations [161,165–167]. Under this view of the hippocampus, temporal and spatial relations are instances of relations which are rich and prevalent—and experimentally accessible—yet not fully comprehensive. This is evidenced by the involvement of the hippocampus in inferring relations that are neither temporal nor spatial [45,162,164,168].

Understanding the hippocampus as intrinsically representing alternatives to actuality suggests that the hippocampus may have a broader role in cognition than is often described. If the role of the hippocampus in cognition is not restricted to particular types of relations such as in time and space, generative neural activity might involve the construction and application of any number of relations across additional domains. In rodents for instance, neural firing in the hippocampus is often studied in relation to space yet can also encode a wide variety of variables from experience such as odours and sounds [100,169–174]. Accordingly, we would expect the hippocampus to exhibit generative activity corresponding to alternatives to actual experience in terms of such variables. This could include linking aspects of experience across modalities into internally constructed representations of possibilities or hypotheticals that have not actually been experienced. In the case of humans, it is particularly notable that the hippocampus has long been linked not only to the acquisition of episodic memory, but of declarative memory more generally, which entails acquisition of semantic memory. By this token, it may be plausible that generative neural activity in humans (in addition to animals) can represent alternatives to actuality by engaging in semantic relations—for instance, in language comprehension or production, and in creativity understood more broadly [33,132,175–178].

This broader view of generativity in the hippocampus may have additional implications at a higher level than representation. An advantage of internal models is that they enable internally directed exploration, or generative simulations and hypothesis formation intended to yield maximum information gain [159]. Interestingly, though such exploration is recognized to be ultimately adaptive, it might have little or no immediate utility, and, further, neural activity implementing this process could be uncorrelated with immediately upcoming behaviour. Exploration can also be driven by curiosity, an intrinsic motivation that has notably been linked to the construction of rich internal models [179]. These are several points of contact between information-based exploration and generative activity patterns. Yet even beyond information-based exploration, it is increasingly recognized that humans and a range of animals can harbour intrinsic motivations expressed as self-determined and self-guided goals, manifesting in behaviour as ‘play’ [180]. Critically, like exploration, play has (practically by definition) little or no immediate utility to subjects, though its relevance or role in advanced cognition is potentially crucial [180]. It is, therefore, worth speculating that analogous play-like adoption of seemingly arbitrary internal aims and constraints is relevant to understanding generative activity patterns in the hippocampus, both in animals and humans. Thus generative hippocampal representations and the hippocampus at large may represent alternatives to actual experience not only to navigate immediately relevant environments and objectives, but also to pursue any number of internally directed and invented goals. Ultimately, doing so may be crucial not only to evolve a greater understanding of past and immediately relevant experience, but also to deal adaptively and flexibly with unexpected scenarios and unknown circumstances in the future.

## 7. Conclusion

Imagination requires the ability to generate experience, thoughts or representations that do not refer to the actual present. This essential ability, termed ‘generativity’, can be understood at the level of the brain and need not entail conscious awareness or mental imagery. Human studies have linked imagination and the construction of hypotheticals to the hippocampus and complementary studies in the rodent hippocampus have identified neural firing patterns corresponding to experiences that do not reflect the actual present. Traditional accounts of hippocampal function often interpret these generative firing patterns, such as those observed during SWR replays and late phases of the theta rhythm, in relation to actual experience in the past and future. This important view may be limited in accounting for the diversity of generative hippocampal firing patterns suggested by recent findings. Rather, we propose that representing alternatives to actual present experience is itself essential to the hippocampus. These representations may span a wide range of self-generated possibilities, hypotheticals and non-actualities of all kinds: from past episodes and anticipated futures, to counterfactuals, alternative presents, novel combinations of experiences and to creative or even playful simulations, including those without spatial or temporal reference. We further suggest that diverse generative hippocampal activity patterns may be used to learn, infer and consider various abstract relations. This in turn would suggest that functions of the hippocampus that refer to time or space (such as episodic memory and mental time travel) may be particular applications of a broader system of imagination (figure 4) [7,15,17,176]. Notably, this view advocates that the function of generative neural activity in the hippocampus may not be characterized by the strength of its correlation to immediate behavioural choices, but rather by its relationships to internal processes [68,113,122,181]. The contribution of generativity to behaviour may not be immediate, and in fact might have an indefinite horizon in the lifetime of subject. This system may be elaborated in humans, supporting frequent and at times seemingly undirected flights of creativity and imagination.

## Data Availability

This article has no additional data.
